# Interaction between position sense and force control in bimanual tasks

**DOI:** 10.1186/s12984-019-0606-9

**Published:** 2019-11-08

**Authors:** Giulia Ballardini, Valentina Ponassi, Elisa Galofaro, Giorgio Carlini, Francesca Marini, Laura Pellegrino, Pietro Morasso, Maura Casadio

**Affiliations:** 1 Department of Informatics, Bioengineering, Robotics, and Systems Engineering, University of Genoa, Genoa, Italy; 2 Italian Institute of Technology, Genoa, Italy

**Keywords:** Proprioception, Matching tasks, Object lifting, Isometric force

## Abstract

**Background:**

Several daily living activities require people to coordinate the motion and the force produced by both arms, using their position sense and sense of effort. However, to date, the interaction in bimanual tasks has not been extensively investigated.

**Methods:**

We focused on bimanual tasks where subjects were required:

- (*Experiment 1*) to move their hands until reaching the same position – equal hand position implied identical arm configurations in joint space - under different loading conditions;
- (*Experiment 2*) to produce the same amount of isometric force by pushing upward, with their hands placed in symmetric or asymmetric positions.

The arm motions and forces required for accomplishing these tasks were in the vertical direction. We enrolled a healthy population of 20 subjects for *Experiment 1* and 25 for *Experiment 2*. Our primary outcome was the systematic difference between the two hands at the end of each trial in terms of position for *Experiment 1* and force for *Experiment 2*. In both experiments using repeated measure ANOVA we evaluated the effect of each specific condition, namely loading in the former case and hand configuration in the latter.

**Results:**

In the first experiment, the difference between the hands’ positions was greater when they were concurrently loaded with different weights. Conversely, in the second experiment, when subjects were asked to exert equal forces with both arms, the systematic difference between left and right force was not influenced by symmetric or asymmetric arm configurations, but by the position of the left hand, regardless of the right hand position. The performance was better when the left hand was in the higher position.

**Conclusions:**

The experiments report the reciprocal interaction between position sense and sense of effort inbimanual tasks performed by healthy subjects. Apart for the intrinsic interest for a better understanding of basic sensorimotor processes, the results are also relevant to clinical applications, for defining functional evaluation and rehabilitative protocols for people with neurological diseases or conditions that impair the ability to sense and control concurrently position and force.

## Background

The ability to lift objects and to apply coordinated forces with both hands and arms is essential for completing several daily living activities. To successfully accomplish ordinary bimanual tasks our Central Nervous System (CNS) has to process the sensory inputs coming from both sides of the body’s midline and coordinates the actions of the two hands, integrating proprioceptive and haptic information.

Asymmetric conditions, such as simultaneously performing different actions with each hand or achieving the same goal in the presence of different sensory inputs from the two sides of the body, might influence task execution in healthy subjects due to cross-modal interference [1–4] as well as impair performance in people suffering from neurological pathologies [5–8]. While bimanual actions have been widely investigated in general terms (e.g. [9–12]), the impact of discordant motion and/or of different forces feedback arising from the two arms has received less attention.

Both position and force sense contribute to efficient neural control of actions that imply interaction with the environment at different levels: they have a role in reflex responses at both spinal and cortical levels, are fundamental for the control of all purposeful movements [13–15] and influence motor learning [16, 17].

Force and motion control have different neural correlates [18–20] and contribute to different action features (e.g. pushing and reaching), but they share neural pathways and sensory receptors [21]. For example, while muscle spindles are known to be mainly responsible for position sense and Golgi tendon organs for force perception, recent studies [22–24] found that muscle spindles are also involved in the perception of force and heaviness. Thus, the simultaneous processing of motions and forces could represent a challenge and it might also lead to reciprocal interferences, a crucial topic that was rather disregarded in recent years [21, 25].

Nevertheless, in the usual formulation of assessment protocols, either in research or clinical environments, position and force sense are mainly evaluated separately, without accounting for their possible interactions or interference [17, 26, 27]. The most commonly used protocols are based on matching tasks, where blindfolded subjects are required to match a reference joint position [21, 26, 28, 29] or a level of muscle contraction [21, 30, 31] with the same or with the other arm, either sequentially or concurrently. These protocols allowed investigating the asymmetries in the upper-limbs position [32, 33] and force [34] control associated with handedness and hand preferences [35, 36]. They were also used to establish indicators for intrinsic cerebral asymmetry at functional and structural levels [31, 37–39] and to find similarity of pathways and sensory receptors between force and position sense [21].

In position matching tasks, few studies demonstrated that changing the sensory inputs affects performance [40–42]. For example, eliminating the antigravity support or adding weights to the reference arm provided an additional position sense cue that improved matching outcomes [41, 42]. However, to our knowledge, this sensory effect has not been evaluated in bimanual tasks with both hands active and engaged toward a common goal. In other words, there is a lack of knowledge on how additional sensory inputs provided symmetrically or asymmetrically to the two hands impact concurrent bimanual control; this is the case for the influence of the loading conditions on position control as well as for the influence of position sense on force control.

More specifically, the purpose of this study was twofold: to investigate how the sense of effort influences the ability to sense and control the position of the hands and to investigate the how the configurations of the arms has impact on the ability to produce isometric force in tasks where the two hands share a common motion or force goal.

Our hypothesis was that asymmetric loading conditions and asymmetric arm configurations might affect, respectively, the accuracy of lifting the two hands at the same height and/or applying bilaterally equal isometric forces. In fact, in mirror symmetric condition the CNS could simply solve the task of guiding the two hands toward the common goal by transmitting the same motor commands to both sides of the body [43–45]. Conversely, in the presence of different sensory feedback from the two arms, the CNS must take into account this difference and compensate for it, producing different bilateral motor commands for achieving the same common goal. We wonder whether the CNS might not account correctly for the mismatch on the sensory inputs between the two limbs when pursuing a bilateral equal force or position goal; the differences in performance among task conditions would highlight this effect.

In order to investigate these hypothesis, we designed and built a device that allowed to implement two bimanual matching experiments: a first experiment investigating position control, in which we requested 20 healthy subjects to place their hands in the same position under different loading conditions; and a second experiment in which 25 healthy subjects had to produce an equal isometric force with the two arms in symmetric or asymmetric configurations. Both tasks were performed without the guidance of a visual feedback.

Preliminary results from this work were presented in abstract form in [46, 47].

## Methods

### Equipment

**Fig. 1:**
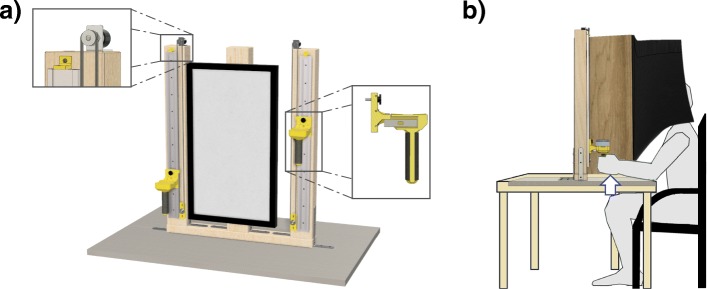
Equipment and experimental set-up. **a** Render of the device with a screen placed in the middle of the two lateral poles, with metal guides where custom-made handles could slide. The motion of each handle was transmitted through a belt and a pulley to a potentiometer that measured its position (left detailed view). Each handle enclosed a load cell (right detailed view) to record the force applied to the handles after fixing them with a screw in specific positions on the guide. The load cells recorded the force applied in the upward direction (i.e., the subjects had to push the handle upward). **b** Experimental set-up. The device was placed on a table and the subjects were seated in front of the screen. A black curtain was attached to the device in order to prevent the visual feedback of their arms [46]. The arrow shows the direction in which the subjects applied the force during the Experiment 2

We designed and built a device (Fig. 1a) for evaluating the ability to control position, force and their interaction in bimanual tasks, as lifting objects and applying controlled isometric forces in the upward direction The device is composed by two robust wooden vertical bars, firmly attached to a base plane. Each bar has a metal linear guide where a custom-made handle can slide or be locked in specific positions. The vertical motion of each handle is transmitted to a potentiometer (Vishay, Malvern, Pennsylvania, USA; maximum resistance of 500 Ω; linearity of ±0.25% FS) via a belt and a pulley in order to provide a precise measurement of the handle position (resolution of 0.27 mm). The friction of the sliding motion of the handle is minimized by a custom-designed bearing block. The handle can be locked in some fixed positions by a mechanical block and in such case the isometric force exert by the subject is measured by a micro load cell (mod. CZL635, Phidgets Inc., Calgary, Canada; full range scale of 5 kg; precision of 0.05% and linearity of 0.05% FS). The analog signals from the potentiometers and the load cells are recorded by a DAQ board (NI USB-6008, National Instruments, Austin, Texas, USA) that is used also to power them. The vertical range of motion of each sliding guide is 0.60 m and the lateral distance of the two guides is 0.50 m, approximately equivalent to the average shoulder-to-shoulder distance.

A screen is placed between the two vertical bars and is used to provide information and instructions to the subjects (see *Experimental set-up and protocol* section for more details).

The handle has a cylindrical shape (90 mm height, diameter of 20 mm) and a weight of 50 g: it is 3D-printed in a rigid and low-weight material (polylactic acid) and cover with high-density foam to increase comfort. It is designed to be easy-to-grasp also by people with low to moderate motor deficits affecting upper limbs or hands [47]. The upper side of the handle terminates with a plate where the experimenter could place additional weights for changing the loading condition during *Experiment 1* (see *Experimental set-up and protocol* section for more details). We used two types of weights, i.e. 250 g or 500 g. Both are shaped as cylindrical containers with the same dimension (30 mm height and diameter of 60 mm): the weight difference is obtained by homogeneously filling the containers with different percentage of clay and lead.

The DAQ board is connected to a laptop via USB. The control software is developed in LabVIEW (National Instrument, Austin, Texas, USA): it acquires the data from the board via an USB channel, samples them at a rate of 100 Hz and send the corresponding visual information to the video screen.

### Experimental set-up and protocol

During the experiments the device was placed on a table and the subjects were seated on a 0.50 m high chair in front of it (Fig. 1b). Subjects grasped the cylindrical part of the device’s handles, maintaining their hands (thumb and index fingers) in contact with the bottom surface of the plates. The distance between the subject and the device was slightly adjusted for each subject, such that their arms were completely extended at the top of the metal guide. The base plane of the device provided a surface where the arms could rest during breaks. A black curtain prevented the visual feedback of shoulders, arms and hands for the entire duration of the experiments. Our goal was to assess proprioceptive ability in terms of position and force control as well as their interaction without visual influence. We designed two separate experiments that required the coordination of the two hands. Each experiment lasted about 30 min; subjects were allowed and encouraged to rest anytime they needed during the execution of each experiment, but they did not ask for any pause. Most of the subjects performed both experiments (see *Subjects* section for more details) and in this case we imposed a break between them to prevent fatigue.

#### Experiment 1: position matching task

**Fig. 2:**
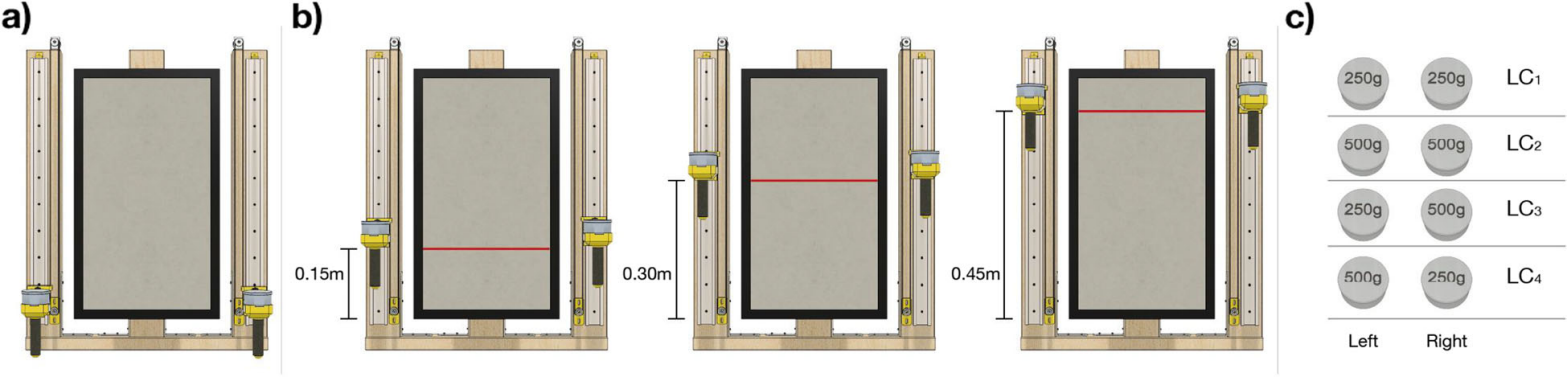
Protocol for Experiment 1. **a** Starting position for the Experiment 1. Every trial started with the handles placed in contact with the base plane. **b** Target positions placed respectively 0.15 m, 0.30 m and 0.45 m above the staring position. The target position was displayed on the screen with a horizontal red line that the subjects had to match bilaterally with the bottom surface of the handle’s plate, which was in contact with their thumb and index fingers. **c** Visual description of the four loading conditions presented during the Experiment 1

During this experiment the handles were free to be moved up and down sliding on the vertical guides. Each trial started with both handles placed in the starting position i.e., with both handles in contact with the base plane (Fig. 2a). Subjects were asked to lift the handles reaching with both hands the same height indicated by a horizontal red line displayed on the screen. The actual positions reached by the two hands were measured when subjects communicated verbally to the experimenter that they had reached the requested target and maintained it for 0.50 s (holding time interval). To evaluate the subjects’ performance we focused on the difference in position between the two hands computed during this holding time interval. We instructed the subjects to reach the required height with both hands, without any additional information, so they could choose the strategy they preferred (see Additional file 1 for more information), without any time constraint.

The visual target line could appear in three different target positions placed respectively at 0.15 m, 0.30 m and 0.45 m from the starting position (Fig. 2b). Two different additional weights (250 g and 500 g) could be placed on top of the left (*L*) and the right (*R*) handles i.e., subjects lifted the two 50 g handles with on top an additional weight. These weights could be equal on the two handles (symmetric loading conditions LC_1_: 250 g; LC_2_: 500 g on both handles) or different (asymmetric loading conditions LC_3_: left = 250 g, right = 500 g; LC_4_: left = 500 g, right = 250 g), for a total of four loading conditions (Fig. 2c). Each loading condition was tested five times for each target position (4 loading conditions * 3 target positions * 5 repetitions) for a total of 60 trials. The loading conditions and the target positions were presented in randomized order. During the test phase, subjects did not receive any feedback about their performance and their hands’ positions.

The experiment included a familiarization phase, prior to the test, during which subjects were required to reach each target position once without any additional weight on the handles. They received a visual feedback about their hands’ position through a black line on the screen, which was connected to the position of the two handles (i.e., the part where the hand was touching the handle). They were aware that in this familiarization phase the task was performed correctly when the black line perfectly overlapped the target red line, but that in the following test the black line would be removed. At the end of the familiarization phase we asked the subjects if they correctly understood the task, otherwise they could extend the familiarization phase.

#### Experiment 2: force matching task

**Fig. 3:**
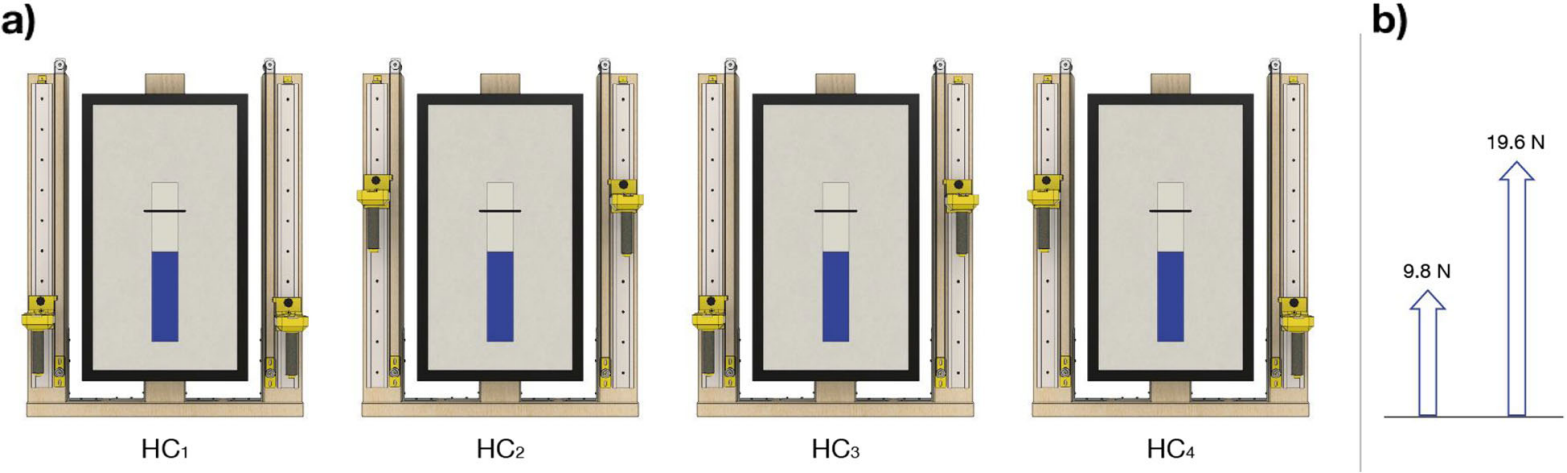
Protocol for Experiment 2. **a** Hand configurations in the Experiment 2 and examples of the real-time visual feedback provided during each trial. The height of the blue bar displayed on the screen was proportional to the sum of the force applied by the two hands. The black line indicated the desired target force that had to be reached with equal force contribution of the two hands. **b** Visual description of the two target forces proposed during the experiment

In this second experiment (Fig. 3), subjects were asked to apply the same amount of isometric force with the two arms pushing up the handles, which were rigidly fixed on the metal guide (Fig. 1a, right detailed view). They had to perform this task with the hand placed in different positions. The subjects did not receive any feedback of the individual hand position and individual hand force. Only the total force level, i.e. the sum of the two hand forces, was explicitly visualized on the video screen as a vertical bar, together with a horizontal line expressing the target level of the total force (Fig. 3a). In this manner it was possible to evaluate the force matching task at different force levels and different hand positions. Two different target force levels were requested: 9.8 N or 19.6 N (Fig. 3b). Two different hand positions were used (0.10 m or 0.30 m above the starting position) for four symmetric/asymmetric hand configurations (Fig. 3a, symmetric HC_1_: 0.10 m, HC_2_: 0.30 m for both hand, or asymmetric HC_3_: L = 0.10 m and R = 0.30 m, HC_4_ vice versa). These four hand configurations were presented five times for each target force in random order (4 hand configurations * 2 target forces * 5 repetitions) for a total of 40 trials. The subjects were instructed to apply the force simultaneously with both hands: if they attempted to do it sequentially, an error message was provided and the trial was discarded. Subjects were also instructed to verbally communicate to the experimenter that they had reached the required amount of force and then they maintained that level of force for 0.50 s (holding time interval). To evaluate the subjects’ performance, we focused on the forces of the two hands measured during this holding time interval. There was no time constrain for completing the trials.

As in *Experiment 1*, there was a familiarization phase before the test. During this phase we provided the subjects with the visual feedback of the force applied by each hand by displaying two lateral bars in addition to the central bar of the total force. Each additional bar had height proportional to the force exerted by the corresponding hand. Subjects were aware that the two additional bars would be not displayed during the test. In the familiarization phase the subjects were asked to perform four of the eight possible combinations of the four hand configurations and two force levels (i.e., each subject experienced all the hand configurations and all the target forces, but not all combinations). The rationale was to minimize the duration of this phase while allowing the subjects to get experience of both arm configurations and both force levels. Then we asked them if they correctly understood the task, otherwise they could extend the familiarization phase.

### Subjects

Twenty subjects participated in both experiments (31 ± 14 years old, 12 females). Five additional subjects participated only to *Experiment 2*, with a total of 25 subjects (30 ± 12 years old, 14 females). Subjects participating in both experiments performed first *Experiment 1*, then *Experiment 2*. We verified that the performance of the twenty subjects performing both experiments were not different from the performance of the other five subjects (repeated-measure ANOVA group effect: *p* = 0.115, all interactions *p* > 0.21), i.e. we did not detect any fatigue effect or carryover effects of *Experiment 1* on *Experiment 2*.

Inclusion criteria were: (i) no evidence or known history of neurological diseases; (ii) normal joint range of motion and muscle strength; (iii) no problems of visual integrity that could not be corrected with glasses or contact lenses, as they could clearly see thetargets that were displayed on the computer screen; (iv) right-hand dominance. All subjects resulted right-handed from the Edinburgh Handedness Inventory [48] (Edinburgh Test Score: 86 ± 17 for the population of the *Experiment 1* and 87 ± 16 for the population of the *Experiment 2*). Each subject signed a consent form to participate in the study and to publish the results of this research. The research and the consent form were conformed to the ethical standards of the 1964 Declaration of Helsinki and approved by the local Ethical Committee.

### Data analysis

Our primary outcome was the systematic difference between the two hands in terms of position in *Experiment 1* and force in *Experiment 2*. On this purpose, we computed two types of *bias-error*, related to position or force control, as the signed difference between the position/force of the two hands, averaged for each subject over the trials performed in the same conditions:

1 $$ \overline{\gamma}=\frac{\sum_{\mathrm{i}=1}^{\mathrm{N}}\ \left({\gamma}_L-{\gamma}_R\right)}{\mathrm{N}} $$

where $\overline{\gamma}$ is the signed difference between the positions reached or the forces applied by the two hands, left (*γ*_*L*_) and right (*γ*_*R*_), during the N trials for the same experimental conditions: target position and loading condition (*Experiment 1*), target force and f hand configuration (*Experiment 2*). This indicator is also a measure of symmetry between the two hands in the two experiments: the lower the error the higher the degree of symmetry.

In addition to the *bias-error*, for each experimental condition we also computed the *variable-error* as the standard deviation of the difference between the two hands at the end of each trial, in terms of position for *Experiment 1* and force for the *Experiment 2*:

2 $$ {\sigma}_{\gamma }=\sqrt{\frac{\sum \limits_{i=1}^N{\left({\gamma}_i-\overline{\gamma}\right)}^2}{N}} $$

this indicator is a measure of performance variability, independent of the degree of correctness of each trial.

Moreover, we computed additional indicators in order to take into account any apparently minor difference between the two matching tasks. In *Experiment 2*, due to the experimental design, the subjects always reached the required target force (i.e., visual feedback of the sum of two forces) and if one hand exceeded half of the target force, the other undershoot it by the same amount. In contrast, the performance of each hand in *Experiment 1* was independent of the other, i.e. one hand could undershoot or overshoot the target position to different extents independently of the behavior of the other hand. Thus, in order to better understand the results of *Experiment 1* we also verified whether each hand overshoot or undershoot the target position by computing the *bias-error* (with Eq. 1) and the *variable-error* (with Eq. 2) of each hand position respect to the target position, namely the ‘*target-bias-error*’ and the ‘*target-variable-error*’. As a final indicator, only for *Experiment 2*, we computed also the *absolute-error*, as the unsigned difference between the forces applied by the two hands averaged for each subject over the trials performed in the same conditions.

### Statistical analysis

Our primary goalwas to assess the influence of:

1. the loading conditions of the two hands on the ability to lift them at the same height in the absence of visual feedback (position control task);
2. the hand configurations on the ability to push upward, applying equal force with the two hands (force control task).

Specifically, using Statistica 7.1 (Statsoft, Tulsa, Oklahoma, USA) we tested in *Experiment 1* the hypothesis that the loading conditions could influence the position sense, whereas in *Experiment 2* we tested the hypothesis that the hand configurations could influence the force applied by the hands. To test both hypotheses we performed a repeated-measures ANOVA (rm-ANOVA) on the two types of *bias-error* with two within-subjects factors: the ‘loading condition’ (4 levels: LC_1,_ LC_2_, LC_3_, LC_4_) and ‘target position’ (3 levels: 0.15, 0.30, 0.45 m) for *Experiment 1*; ‘hand configuration’ (4 levels: HC_1,_ HC_2_, HC_3_, HC_4_) and ‘target force’ (2 levels: 9.8, 19.6 N) for *Experiment 2*. A significant effect of the first factor in each experiment would support our hypotheses. To further understand our outcomes, we applied the same analysis to the *variable-error* in both experiments and to the *absolute-error* only in *Experiment 2.*

Moreover, to evaluate to what extent the two hands matched the target positions in *Experiment 1,* we performed a rm-ANOVA on the *target-bias-error* and *target-variable-error* with two within-subjects factors: ‘hand’ (2 levels: right and left) and the ‘loading condition’ (4 levels: LC_1,_ LC_2_, LC_3_, LC_4_).

We verified the normality of the data using Lilliefors test. All data were normally distributed. We tested for the sphericity of the data using Mauchly’s test and the Greenhouse-Geisser correction was applied when the assumption of sphericity was rejected. Specifically, the sphericity assumption was verified for all indicators, except for the *bias-error* in *Experiment 1* (target position factor: Chi-squared: χ^2^ = 8.70, Greenhouse-Geisser epsilon: ε_G-G_ = 0.72; loading condition factor: χ^2^ = 20.58, ε_G-G_ = 0.60). We performed a post-hoc analysis (Fisher’s LSD test) to further investigate statistically significant main and interaction effects. Statistical significance was set at the family-wise error rate of α = 0.05. The *p*-values are reported without the correction for multiple comparisons, however we verified that the significant results were robust to Bonferroni-Holm corrections and we reported in the text when it was not.

## Results

All subjects successfully participated in this study and did not report any adverse event in terms of muscle aches, fatigue or misunderstanding of the tasks.

### Experiment 1: position matching task

**Fig. 4:**
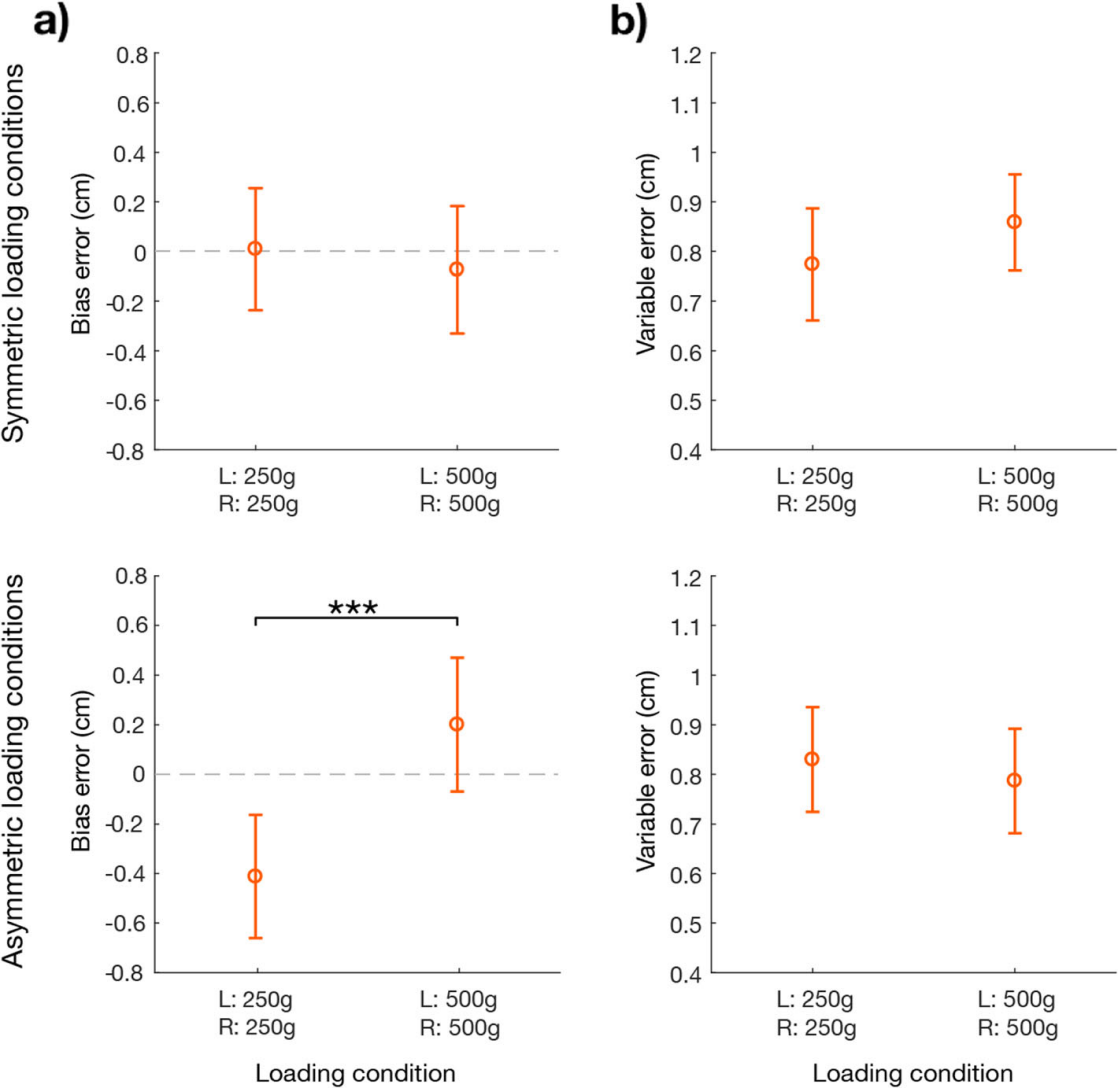
Experiment 1. Indicators of performance in the position matching task: difference between the heights reached by the two hands (left (L) hand – right (R) hand) with respect to the loading conditions (x axis) averaged over the three target positions. Top row: symmetric loading conditions (LC_1_ and LC_2_). Bottom row: asymmetric conditions (LC_3_ and LC_4_). All the panels show the population results (mean value ± SE). Panel **a** bias-error. The dashed line indicates no difference between the two hands (i.e., the desired performance). Panel **b** variable-error. * indicates statistical significance: * *p* < 0.05, ** *p* < 0.01 and *** *p* < 0.001

The *bias-error* was influenced by the loading condition (loading condition effect: F (3, 57)=13.47; *p* < 0.001), regardless of the target position (target position effect: F (2, 38)=1.67; *p* = 0.210; interaction target x load effect: F (6,114) = 1.366; *p* = 0.234). Indeed, in the symmetric loading conditions (Fig. 4a, top row) the *bias-error* was close to zero and there was not a statistical difference in height between two hands when both held either lighter (250 g) or heavier weights (500 g) (post-hoc analysis: LC_1_-LC_2_: *p* = 0.403). Conversely, a significant difference (post-hoc analysis: LC_3_-LC_4_: *p* < 0.001) emerged between the two asymmetric conditions (Fig. 4a, bottom row): the hand with the lighter weight reached systematically a lower height with respect to the hand with the heavier weight, as indicated by the different sign of the *bias-error* of LC_3_ and LC_4_. The *bias-error* was more marked when left hand had the lighter weight, i.e. in LC_3_, in fact this condition was significantly different from all the other three (*p* ≤ 0.001 in all cases). In LC_4,_ i.e., when the lighter weight was on the right hand, the *bias-error* changed sign with respect to LC_3_, but its absolute value was lower. The difference between LC_4_ and LC_2_ was statistically significant (post-hoc analysis: LC_2_-LC_4_: *p* = 0.007) while the difference between LC_4_ and LC_1_ did not, but it was close to the threshold of significance (post-hoc analysis: LC_1_-LC_4_: *p* = 0.058). Neither the loading condition nor the target position had a significant effect on the *variable-error* computed for the difference in height between the two hands (*p* > 0.05 for both the effects) (Fig. 4b).

**Fig. 5:**
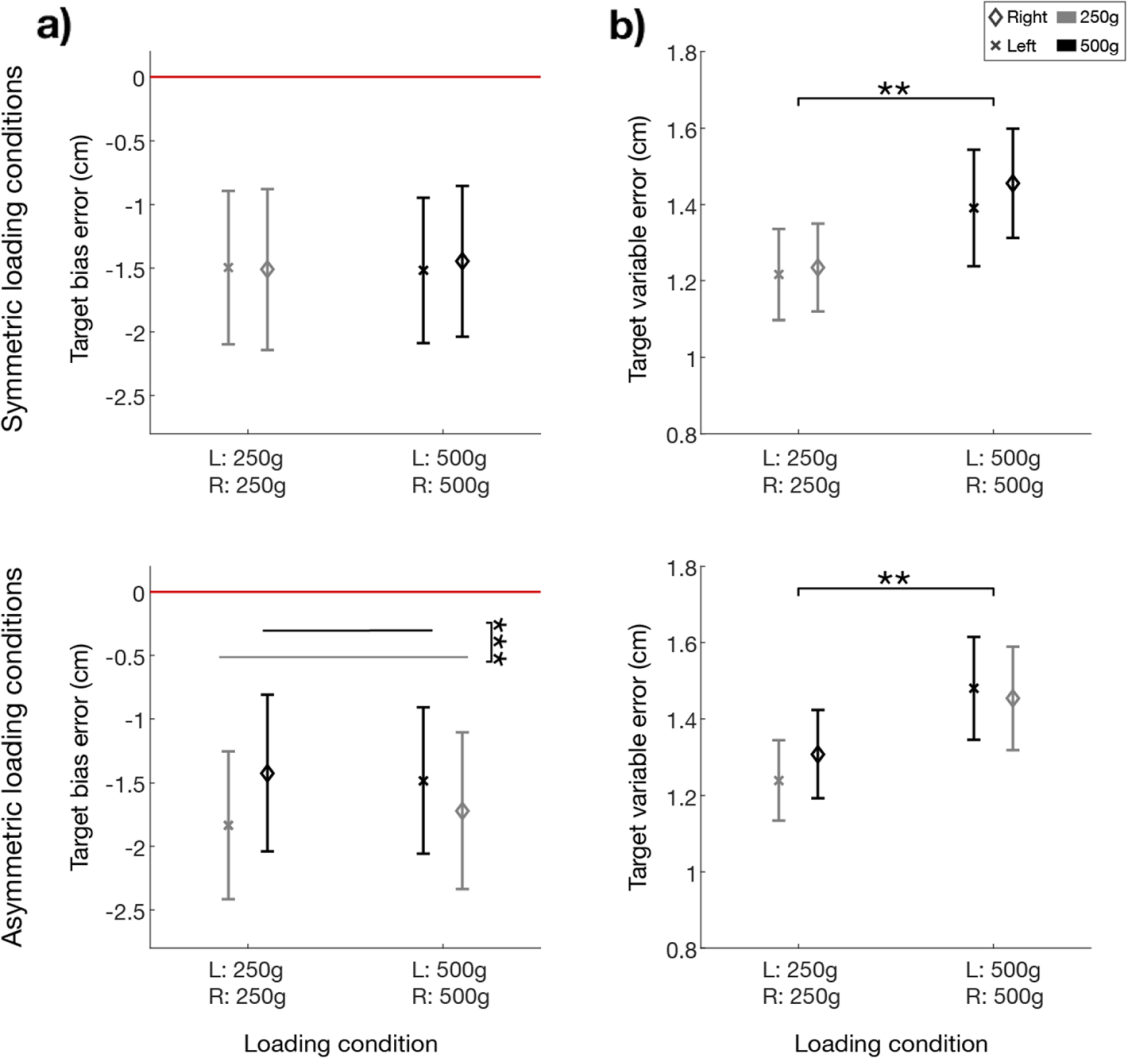
Experiment 1. Indicators of performance in the position matching task with respect to the target position: difference between the heights reached by each hand and the target position displayed with respect to the loading conditions (x axis) averaged over the three target positions. Top row: symmetric loading conditions (LC_1_ and LC_2_). Bottom row the asymmetric conditions (LC_3_ and LC_4_). All the panels show the population results (mean value ± SE). The left hand is represented by a ‘x’ symbol, right hand by a ‘diamond’ symbol. Panel **a** target-bias-error. The red line represents the target position. Panel **b** target-variable-error. Colors indicates the loading conditions of the hand: gray is for the lighter weight (250 g) and black for the heavier (500 g). * indicate statistical significance: * *p* < 0.05, ** *p* < 0.01 and *** *p* < 0.001

To further understand the effect of the loading condition, we analyzed also the difference between each hand and the target position. The *target-bias-error* highlighted that both hands in all the conditions undershoot the target position (1.55 mean ± 0.60 SE cm, Fig. 5). This undershoot was equal for the two hands in both the symmetric conditions (Fig. 5a top row). Conversely, in the asymmetric conditions (Fig. 5a bottom row) this undershoot increased for the hand that held the lighter weight, i.e. the left in LC_3,_ and the right in LC_4,_ determining a highly significant hand x loading condition effect: F (3, 57)=14.94; *p* < 0.001. More specifically, the hand with lighter weight reached a significantly lower height with respect to the contralateral hand in the asymmetric conditions and also with respect to the height reached by both hands in symmetric conditions; *p* < 0.005 in all cases.

The *target-variable-error* (Fig. 5b), instead, revealed only a significant difference across the loading conditions (loading condition effect: F (3, 57)=5.19; *p* = 0.003). Specifically, the *target-variable-error* was lower in LC_1_ and LC_3_ with respect to LC_2_ and LC_4,_ i.e. the variability of the height reached by both hands was lower when the left hand held a lighter weight (post-hoc analysis: LC_1_-LC_2_: *p* = 0.008; LC_1_-LC_4_: *p* = 0.001; LC_2_-LC_3_: *p* = 0.042; LC_3_-LC_4_: *p* = 0.009, with the significance for LC_2_-LC_3_ not robust to Bonferroni-Holm correction; other comparisons *p* > 0.50).

### Experiment 2: force matching task

**Fig. 6:**
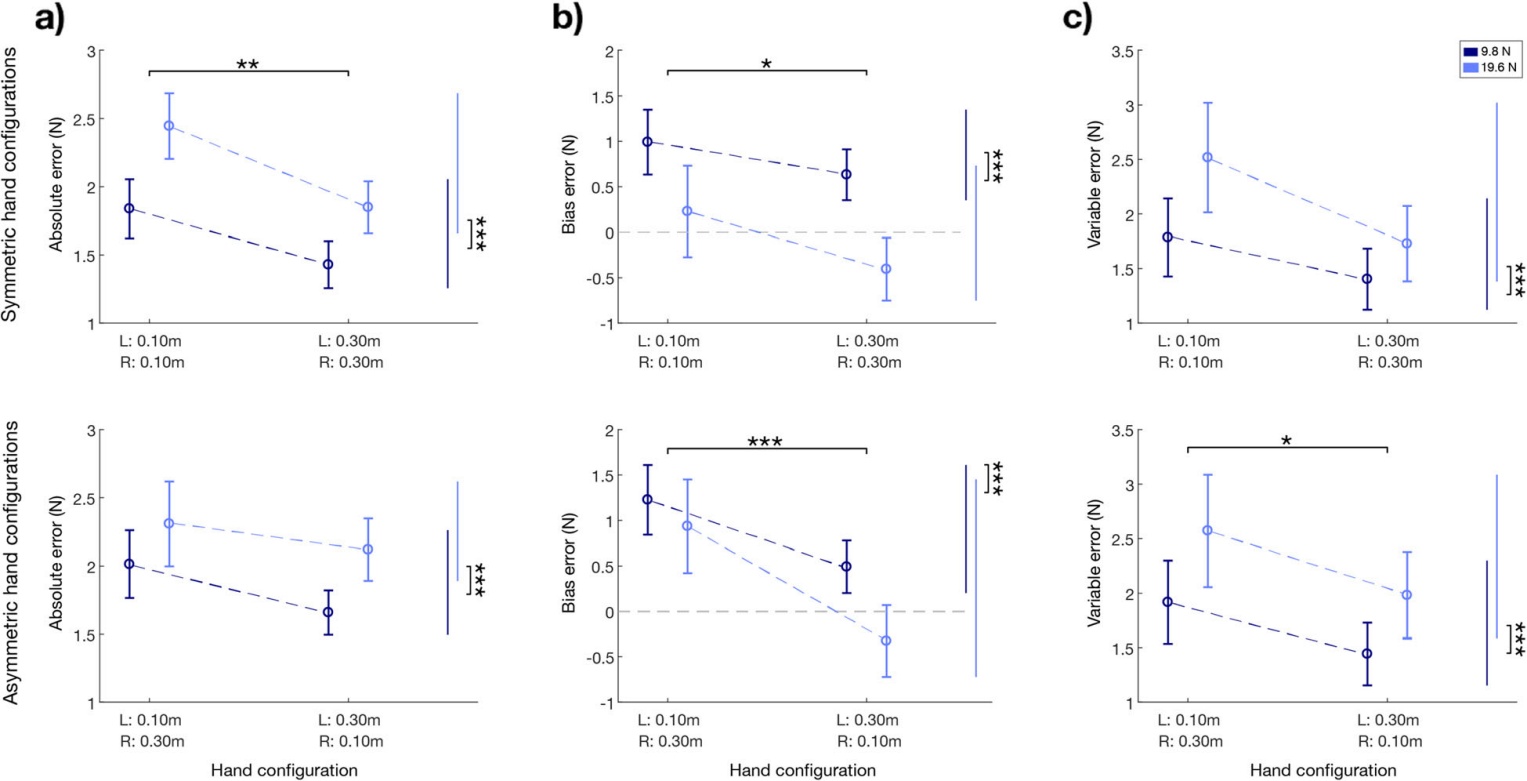
Experiment 2. Indicators of performance in the force matching task: difference between the forces applied by the two hands (left (L) hand – right (R) hand) displayed with respect to hand configurations averaged over the two target forces. Top row: symmetric hand configurations (HC_1_ and HC_2_). Bottom row: asymmetric hand configurations (HC_3_ and HC_4_). All the panels show the population results (mean value ± SE). In each panel, the data is reported separately for each target force (dark blue for the lower force, light blue for the higher) and the dashed lines connect the performance in the different hand configurations for the same target force. Panel **a** absolute-error. Panel **b** bias-error, here the gray dashed line represents the null difference between the two hands (i.e., the desired performance). Panel **c** variable-error. * indicates statistical significance: * *p* < 0.05, ** *p* < 0.01 and *** *p* < 0.001

The *absolute-error* (Fig. 6a) computed as the absolute difference between the left and right hand in terms of applied force was influenced by two factors:

- ➣ the amount of total force applied by the subject (target force effect: F (1, 24)=9.11; *p* = 0.006), i.e., higher force corresponded to higher *absolute-error*;
- ➣ the hand configuration (F (3,72) = 4.22; p = 0.008), i.e., the left hand in the lower position corresponded to higher *absolute-error* (left hand at lower vs higher position: F (1,99) = 12.25; p = 0.001)

The first factor was due to the *variable-error* i.e. higher target force led to higher *variable-errors* (target force effect: F (1, 24)=30.36; *p* < 0.001). Instead, the *bias-error* had an opposite and significant behavior: the systematic difference between the two hands was lower for the higher target force (target force effect: F (1, 24)=15.67; *p* < 0.001, no interaction effects were observed *p* > 0.11 in all cases).

Conversely, the second factor was due mainly to the systematic component of the error, i.e. to the *bias-error* (hand configuration effect: F (3,72) = 6.72; *p* < 0.001; left hand at lower vs higher position: F (1,99) = 20.63; *p* < 0.001; Fig. 6b), while the *variable-error* had the same trend without reaching the significance threshold (hand configuration effect: F (3,72) = 2.14; *p* = 0.102; Fig. 6c). This effect for the *bias-error* was significant in both the symmetric and asymmetric configurations (post-hoc analysis: HC_1_-HC_2_: *p* = 0.028; Fig. 6b upper panel, HC_3_-HC_4_: *p* < 0.001; Fig. 6b bottom panel), indicating that when the left hand was in the lowest position, it applied systematically more force than the right hand, independently of the position of the contralateral hand. This overshoot remarkably decreased when the left hand was in the highest position, to the point that for the higher target force, the trend was inverted: the right hand applied more force than the left. The *absolute-error*, when we considered the two conditions separately, was statistically significant only for the symmetric hand configurations (post-hoc analysis: HC_1_-HC_2_: *p* = 0.001; Fig. 6a upper panel, HC_3_-HC_4_: *p* = 0.114; Fig. 6a bottom panel). For all the parameters no significant differences were found between symmetrical and asymmetrical hand configurations (*p* > 0.10).

## Discussion

We designed two experiments: in *Experiment 1*, the subjects had to lift both hands at the same height under different loading conditions; in *Experiment 2*, they had to apply equal isometric forces in the upward direction, with the hands in different positions. In both cases the subjects could perform the matching task without relying on visual feedback, but only on proprioception. Our hypotheses were that:

- ➣ asymmetric loadings, i.e. different weights held by the two hands, would affect bimanual position control in *Experiment 1*;
- ➣ asymmetric configurations of the hands/joints would influence bilateral force control in *Experiment 2*.

For both conditions we expected decreased performance with respect to the corresponding symmetric ones.

The results confirmed the first hypothesis, demonstrating that an asymmetric loading condition determined a systematic *bias-error* between the heights reached by the two hands. The target height did not influence the performance, as expected for our population of healthy subjects. Conversely, the second hypothesis had to be rejected because the results showed that the configuration of the hands affected the ability to apply the desired bimanual force, but this effect was dominated by the left hand position, regardless of the position of the right hand.

### Experiment 1: position matching task

The reported ability to lift both hands at the same height in symmetric loading conditions is probably due to an underlying synchronization tendency between the hands, well established in several bimanual tasks [49–52]. Indeed, in this experiment temporal and spatial parameters constrain the limb movements, [43, 52, 53], inducing intermanual coordination and leading to a systematic bias toward similar patterns [54, 55].

In the asymmetric conditions, the hand holding the lighter weight reached a position farther from the target, i.e., had a higher *target-bias-error* than the other hand. This is in agreement with previous findings suggesting that the effort required to hold a limb against the force of gravity or a weight in static condition provide a positional cue that improves performance in upper limb joint matching tasks [40–42]. Moreover, holding heavier weights increases muscular activation of the same muscle groups [56, 57], determining a higher proprioceptors’ activation [56], that leads to better performance in position matching tasks [31].

However, in the symmetric loading conditions we did not find any significant difference between the trials in which both hands held heavier or lighter weights . Thus, in our experiment based on additional light-weights but with a marked relative difference between them,^1^ the position control was influenced by the different weights, only when they were unbalanced, i.e. in asymmetric loading conditions, but not when they were balanced, i.e. in symmetric loading conditions.

As for the *variable-error*, the two hands were coupled, i.e. the variability of the two hands with respect to their average error was similar in all the conditions. Specifically, in symmetric conditions (LC_1_ and LC_2_) both hands had higher *target-variable-error* when holding heavier than lighter weights. In symmetric conditions the two hands received the same additional feedback (i.e., the position of the other hand) and since the variability associated with force/heaviness perception is known to be higher for higher forces/weights [44, 45], we expected the two hands having higher variability when holding heavier weights. In the two asymmetric conditions (LC_3_ and LC_4_), the two hands received a different feedback in dependence of the weight they were holding. If the CNS when controlling one hand was unable to integrate the information of the other hand, each hand would maintain higher *target-variable-error* when holding the heavier weight. However, the latter was only the behaviour of the left hand, but not of the right hand, that instead modified its behaviour, matching the performance of the left hand. Thus, the CNS when controlling the right hand is integrating and accounting for the information coming from the left hand holding a different weight. Conversely, when controlling the left hand, the CNS did not account for the feedback from the right hand, relying only on the left hand’s proprioceptive information. This result suggests a ‘leading role’ of the left since the variability of the two hands was coupled in all conditions, independently of the weight hold by each hand, and this behaviour seems to be determined by the left hand, at least in right-handed subjects.

The dominant role in proprioceptive tasks of the left hand has been previously reported in the literature [36, 61] and also the results of the second experiment, discussed in next paragraph, supported this conclusion.

### Experiment 2: force matching task

The force outcomes mainly depended on the position of the left hand, regardless of the right hand, i.e. for this bimanual isometric force task we found a leading role of the left hand and not an effect of hand configuration symmetry.

This result is surprising and in apparent contrast with the initial assumption that the equal position of the two hands would imply better performance as it corresponded to equal joint configurations that require a similar motor commands for the two sides of the body.

However, this paradoxical result may be explained by the dichotomous model, firstly proposed in [36]. The model suggests that the upper limbs’ differences found in the behavioral performances are based on the different key sources of movement-related sensory feedback, which they rely more on: vision or proprioception. According to such view, during bimanual activities the dominant (right) arm relies more on visual feedback, whereas the non-dominant (left) arm is better off with proprioceptive feedback [36, 61]. Thus, in our experimental paradigm, where the task could not be solved relying on visual feedback, the left arm may be advantaged and consequently assume a leading, dominant role. A further support to this interpretation comes from another similar study demonstrating that the non-dominant limb is specialized in controlling static exertion of forces [3, 62, 63].

Another result worth consideration is that the errors, and especially the *bias-error*, were lower when the left hand was in the higher position. We may speculate that the better performance of the leading hand in the highest position could be due to muscular activation. In particular, for exerting the required isometric forces, subjects may need to recruit motor units at the shoulder/trunk level when the hand is in the higher position compared to when it is in lower position: this may imply an increase of the motor commands to produce the same amount of force that could lead to a better force perception [34, 64, 65], explaining the lower errors. Notice also that in different arm configurations, different muscle groups contribute to produce the same level of force, thus the variability (i.e., *variable-error*) of the resulting force could change depending on which specific muscles are recruited and how they are activated: in our experiment the recruitment of shoulder and trunk muscles determined also a decrease on variability. For different levels of force, the results confirmed that *variable-error* depended on the applied force: the variability increased when higher force was required. Indeed, for biological signals it is common to have larger variability associated with higher amplitude of the signals, and it is well known for force applied by the fingers or in unimanual tasks [4] as well as for bimanual matching tasks [43, 44].

The magnitude of the desired force had also another relevant effect: the left hand applied more force than the right hand for the lower target force, but for the higher target force such effect was decreased and even inverted. This result is consistent with the literature about sequential matching tasks: the force applied by the left hand is significantly altered by the amount of the required force [31].

### Limitations and future directions

A concurrent acquisition of muscle signals was not performed. This could allow a deeper understanding of the neural mechanism underling our results, providing further support for the explanations proposed in the *Discussion*. Thus, future studies might focus on recording surface electromyographic data, especially to further investigate the relation between the number of recruited muscle fibers and proprioceptive errors. Specifically, we will aim at testing with the set-up of *Experiment 1* a wider range of weights since the results we found could be valid only for lighter and not for heavier weights, due to not linear relations between proprioceptive errors and muscle fiber activations. As for *Experiment 2*, there are two unaccounted, potentially relevant factors that could have influenced our results: tactile feedback and grip force. Thus, our future studies will focus on investigating their role on force matching task, also by modifying the material of the handles’ cover and by adding supplemental somatosensory feedback.

## Conclusions

From our experiments on the interaction between position sense and sense of effort in bimanual tasks on healthy subjects emerged that the position sense is influenced by the load applied to the hands and the interference is evident when the load had different weights. Conversely, the accuracy of the sense of effort, was not affected by symmetric/asymmetric arm configurations, but was specifically determined by the position of the left arm (for our right-handed subjects), regardless of the right arm position, clearly indicating the leading role of the left hand for the accurate perception of interaction forces.

In spite of the limitations of the experimental design^2^ we feel that the results provide an informed starting point for approaching the broader issue of sensory-motor interactions while offering a pathway for the clinical assessment and rehabilitation of neuromotor deficits. Adding electromyographic analysis of the recruited muscle to the kinematic/kinetic analysis of the current setup and investigating the effect of grip force and somatosensory feedback are also promising future developments that we plan to pursue.

### Implication for functional evaluation and rehabilitation

These results are relevant for clinical evaluations and rehabilitative applications. In fact, while providing new insights about the interaction between force and position control in healthy individuals, they can be also used to define a quantitative evaluation of proprioception in bilateral tasks for people with neurological disorders and stroke survivors. For example, recently with this device and a simplified version of this protocol, we tested bilateral position and force deficits and asymmetries in people with Multiple Sclerosis [47]. Moreover, the device can be used to train subjects to perform symmetric movements and to apply simultaneously equal forces with the two arms in the upward direction. To this end we are currently working also on a motorized version of this device.

## Supplementary information

**Additional file 1.** Analysis of the movement and force strategies applied to solve the task. We analyzed the strategies used by the subjects for accomplishing the tasks, to verify if they can provide further explanations of the results presented in the manuscript. In Experiment 1 we found that the loading conditions influenced the kinematic strategy during the position matching task. In Experiment 2 the strategy adopted for bimanual force exertion was not influenced by symmetric/asymmetric arm configurations, but by handedness or hand preference effects. **Figure S1.** Example of speed and force profile.

## Data Availability

The datasets used and/or analyzed during the current study are available from the corresponding author on reasonable request.

## Abbreviations

*HC*_1_-*HC*_2_-*HC*_3_-*HC*_4_: Hand configurations used during the *Experiment 2*
*JND*: Just Noticeable Difference
*L*: Left hand
*LC*_1_-*LC*_2_-*LC*_3_-*LC*_4_: Loading conditions used during the *Experiment 1*
*R*: Right hand
rm-ANOVA: Repeated measures ANOVA
*SE*: Standard error

## Footnotes

1 The Just Noticeable Difference (JND), i.e., the minimum percent variation between two stimuli necessary to perceive a difference [58], was measured for force perception in lifting task yielding a rough 10% figure [58–60]. Therefore, the subjects of our experiments, although holding light-weights operated well beyond such threshold, with one weight being 83% heavier than the other.

2 (i) restricting the analysis to a single axis (the vertical axis) and (ii) focusing on steady-state (static positions and isometric forces) rather than dynamics.
