# Supplementary material for: Interaction between position sense and force control in bimanual tasks
Source: J Neuroeng Rehabil. 2019 Nov 8;16:137. doi: 10.1186/s12984-019-0606-9 (PMC6839077; doi:10.1186/s12984-019-0606-9)
Supplement: Supplementary file 1 — Additional file 1. Analysis of the movement and force strategies applied to solve the task. We analyzed the strategies used by the subjects for accomplishing the tasks, to verify if they can provide further explanations of the results presented in the manuscript. In Experiment 1 we found that the loading conditions influenced the kinematic strategy during the position matching task. In Experiment 2 the strategy adopted for bimanual force exertion was not influenced by symmetric/asymmetric arm configurations, but by handedness or hand preference effects. Figure S1. Example of speed and force profile. [file 12984_2019_606_MOESM1_ESM.zip › 12984_2019_606_MOESM1_ESM.docx]

***Analysis of the movement and force strategies applied to solve the task***

Subjects were free to choose the strategy that they preferred for accomplishing the tasks without any time constrain. We identified and analyzed the chosen strategies in order to understand to which extent they can provide further explanations of the results presented in the manuscript.

In the *Experiment 1* we analyzed the speed profiles of the two hands in each trial and found that they are characterized by a single dominant peak and by a series of small peaks (e.g. Figure A1a). We could then divide the movements in a ballistic phase followed by an adjustment phase. In particular, the ballistic phase initiated when the speed exceeds the threshold of 10% of the first peak and terminates when it crosses downward the same threshold for the first time. The adjustment phase went from this latter instant to the end of the holding time (see *Methods* section). For each phase we computed the duration and the mean speed. In the ballistic phase we also computed the amplitude and the time of the peak. The onset time of the motion was computed in two ways, i.e., as the time instant when the speed profile of each hand exceeded (1) 10% of its peak (relative threshold) and (2) an absolute threshold of 2.8 cm/s^[[1]](#footnote-1)^. In the adjustment phase we computed the standard deviation of the speed profile.

In *Experiment 2* we analyzed the force profiles separately for two hands, considering as starting time of each trial the instant when the force applied by both hands exceeded for the first time the threshold level of 0.50 N. The force profiles were characterized by a first phase in which they reached a main peak, and a following adjustment phase in which the force level remained high (e.g. Figure A1b). We computed the amplitude and the timing of the first peak. We considered that the adjustment phase started with the first minimum of the force profile after the main peak and ended with the holding time (see *Methods* section). For this phase we computed the duration and the amplitude of the force profile, characterizing the latter with the standard deviation (std force adjustment) and the corresponding coefficient of variance (CV), i.e. the standard deviation divided by the mean value.

FIGURE A1 HERE

**Statistical analysis**

For the *Experiment 1* we performed a rm-ANOVA with three within-subjects’ factors: ‘hand’ (2 levels: left, right), ‘target position’ (3 levels: 0.15 m, 0.30 m and 0.45 m above the starting position) ‘weight’ (4 levels: the additional weights added on one hand (250 g and 500 g) considered both in symmetric and asymmetric loading conditions, i.e., when the other hand hold the same or different weights).

For *Experiment 2* we performed a rm-ANOVA using three within-subjects’ factors: ‘hand’ (2 levels: left, right), ‘hand configuration’ (4 levels: HC_1,_ HC_2_, HC_3_, HC_4_), and ‘target force’ (2 levels: 9.81 N, 19.62 N).

We verified the normality of the data using Lilliefors test. All data were normally distributed. We tested for the sphericity of the data using Mauchly’s test and the Greenhouse-Geisser correction was applied when the assumption of sphericity was rejected. This happened in *Experiment 1* for the time (weight factor: Chi-squared: χ^2^=16.24, Greenhouse-Geisser epsilon: ε_G-G_=0.63; target factor: χ^2^=16.68, ε_G-G_=0.62) and the amplitude (weight factor: χ^2^=15.72, ε_G-G_=0.65; target factor: χ^2^=9.07, ε_G-G_=0.72) of the peak velocity, in the *Experiment 2* for the CV (hand configuration factor: χ^2^=11.97, ε_G-G_=0.80) and for the duration of the adjustment phase (hand configuration factor: χ^2^=23.09, ε_G-G_=0.60).

We also performed a post-hoc analysis (Fisher’s LSD test) to further investigate statistically significant main effects. Statistical significance was set at the family-wise error rate of α = 0.05. The p-values are reported without the correction for multiple comparisons, however we verified that the significant results we reported were robust to Bonferroni-Holm corrections and we reported in the text when they were not.

**RESULTS**

***Experiment 1.*** The two hands rose concurrently, i.e., no statistically significant difference between the two hands in the peak time as well as in the onset time computed in both ways (all p>0.18). Also all the other parameters (Table 1) were similar for the two hands (all p>0.07; Table 2).

Conversely, all parameters depended on the weight factor (Table 1), except for the duration of the adjustment phase (F(3,57)=1.93, p=0.134). In the symmetric conditions, in presence of heavier weights on both hands, the mean speed computed from the movement onset to the end of the adjustment phase was significantly lower than in presence of lighter weights. This behavior is the same for all the speed parameters. Indeed, in the ballistic phase the peak amplitude and the mean speed were lower holding the heavier weight as well as the standard deviation and the mean speed in the adjustment phase. The duration of both phases was longer. Instead in the asymmetric conditions the difference between the speed of the hands holding heavier or lighter weight, decrease assuming intermediate values with respect the two symmetric conditions for all the parameters, except for peak time, and for the duration of the adjustment phase. Comparing symmetric and asymmetric conditions, we found that the hand holding 250 g had a delayed velocity peak in the asymmetric condition, while for the heavier weight there was no change.

As expected all the parameters differed for the target positions, (p≤0.005 in all cases), except for the duration of the adjustment phase (F(3,57)=0.27, p=0.764).

**Table 1.** Mean ± standard error for the ‘Position strategy parameters’ in *Experiment 1* averaged over the three target positions reported for the left hand (first row) and the right hand (second row)

|  |  | 250 SYM | 500 SYM | 250 ASYM | 500 ASYM |
| --- | --- | --- | --- | --- | --- |
| PEAK AMPLITUDE (cm/s) | Left | 30.9 ± 1.6 | 26.2 ± 1.5 | 27.8 ± 1.6 | 28.3 ± 1.6 |
|  | Right | 30.1 ± 1.6 | 25.3 ± 1.3 | 27.5 ± 1.6 | 27.4 ± 1.7 |
| PEAK TIME (s) | Left | 1.38 ± 0.04 | 1.53 ± 0.06 | 1.46 ± 0.07 | 1.52 ± 0.05 |
|  | Right | 1.35 ± 0.03 | 1.52 ± 0.07 | 1.41 ± 0.04 | 1.50 ± 0.05 |
| ONSET TIME (s) | Left | 0.73 ± 0.01 | 0.72 ± 0.01 | 0.68 ± 0.01 | 0.78 ± 0.01 |
|  | Right | 0.72 ± 0.01 | 0.74 ± 0.01 | 0.70 ± 0.01 | 0.78 ± 0.01 |
| ONSET TIME ABSOLUTE TH (s) | Left | 0.73 ± 0.02 | 0.78 ± 0.03 | 0.74 ± 0.03 | 0.81 ± 0.02 |
|  | Right | 0.74 ± 0.02 | 0.81 ± 0.04 | 0.73 ± 0.02 | 0.82 ± 0.02 |
| MEAN SPEED (cm/s) | Left | 11.7 ± 1.0 | 9.6 ± 0.9 | 10.2 ± 1.1 | 10.6 ± 0.9 |
|  | Right | 11.5 ± 1.0 | 9.4 ± 0.8 | 10.4 ± 0.9 | 10.3 ± 1.1 |
| BALLISTIC DURATION (s) | Left | 1.12 ± 0.14 | 1.47 ± 0.16 | 1.34 ± 0.17 | 1.24 ± 0.14 |
|  | Right | 1.22 ± 0.14 | 1.53 ± 0.18 | 1.36 ± 0.16 | 1.43 ± 0.20 |
| BALLISTIC MEAN SPEED (cm/s) | Left | 20.5 ± 1.2 | 17.2 ± 1.0 | 18.3 ± 1.3 | 18.9 ± 1.1 |
|  | Right | 19.9 ± 1.3 | 16.5 ± 1.0 | 18.2 ± 1.2 | 18.0 ± 1.3 |
| STD VELOCITY ADJUSTMENT (cm/s) | Left | 5.8 ± 0.6 | 4.2 ± 0.4 | 4.6 ± 0.4 | 5.3 ± 0.5 |
|  | Right | 5.2 ± 0.5 | 4.0 ± 0.4 | 4.6 ± 0.4 | 4.8 ± 0.5 |
| ADJUSTMENT DURATION (s) | Left | 4.88 ± 0.55 | 4.95 ± 0.54 | 5.08 ± 0.57 | 5.11 ± 0.58 |
|  | Right | 4.79 ± 0.55 | 4.87 ± 0.52 | 5.07 ± 0.57 | 4.89 ± 0.53 |
| ADJUSTMENT MEAN SPEED (cm/s) | Left | 2.9 ± 0.3 | 2.2 ± 0.2 | 2.4 ± 0.2 | 2.67± 0.3 |
|  | Right | 2.6 ± 0.3 | 2.2 ± 0.2 | 2.4 ± 0.2 | 2.6 ± 0.3 |

**Table 2.** F and p values of the significant ‘Position strategy parameters’ in *Experiment 1*

|  | HAND | | WEIGHT | | TARGET POSITION | |
| --- | --- | --- | --- | --- | --- | --- |
|  | F(1,19) | p | F(3,57) | p | F(2,38) | p |
| PEAK AMPLITUDE | 1.72 | 0.205 | 29.38 | <0.001 | 167.81 | <0.001 |
| PEAK TIME | 1.31 | 0.267 | 11.75 | <0.001 | 8.72 | 0.005 |
| ONSET TIME | 1.14 | 0.300 | 53.67 | <0.001 | 27.25 | <0.001 |
| ONSET TIME ABSOLUTE TH | 0.714 | 0.409 | 10.34 | <0.001 | 4.74 | 0.015 |
| MEAN SPEED | 0.14 | 0.715 | 13.25 | <0.001 | 91.30 | <0.001 |
| BALLISTIC DURATION | 1.63 | 0.217 | 23.10 | <0.001 | 19.88 | <0.001 |
| BALLISTIC MEAN SPEED | 1.51 | 0.237 | 19.86 | <0.001 | 162.77 | <0.001 |
| STD SPEED ADJUSTMENT | 3.48 | 0.078 | 16.95 | <0.001 | 39.92 | <0.001 |
| ADJUSTMENT DURATION | 2.42 | 0.136 | 1.93 | 0.134 | 0.27 | 0.764 |
| ADJUSTMENT MEAN SPEED | 1.49 | 0.237 | 13.34 | <0.001 | 37.17 | <0.001 |

***Experiment 2.*** Almost all the parameters (Table 3) computed on the force profiles were significantly different between the two hands (Table 4). In particular the left hand had a higher (F(1,24)=9.52, p=0.005) and delayed (F(1,24)=5.43, p=0.028, not robust to Bonferroni-Holm corrections) force peak with respect to the right hand. In the adjustment phase the left hand had a longer duration (F(1,24)=5.20, p=0.032, not robust to Bonferroni-Holm corrections) and lower CV (F(1,24)=14.43, p=0.001), but the latter was due to the higher mean values of the force since the std was equal between the two hands (p>0.54). Also the level of the target force influenced significantly these parameters. Specifically, an higher target force induced a delay in the peak force (F(1,24)=183.13, p<0.001), a longer duration of the adjustment phase (F(1,24)=14.20, p=0.001), and a lower CV (F(1,24)=6.13, p=0.021, not robust to Bonferroni-Holm corrections). Also in this case~~s~~ the latter was due to a higher mean value.

The force peak was not different in amplitude when comparing the hand configurations (F(1,24)= 2.50, p=0.066), but its timing was significantly higher when both hands were at the lower position respect to all the other conditions (all p<0.02). Both the CV and the std of the force during the adjustment phase had a significant difference based on the hand configurations (CV: F(3,72)=7.35, p=0.001; std: F(3,72)=10.10, p<0.001), specifically both parameters were higher in the HC_4_ , i.e. when the left hand was higher than the right hand, with respect to all the other hand configurations (Table 3).

**Table 3.** Mean ± standard error of the ‘Force strategy parameters’ in *Experiment 2* averaged over the two target forces reported for the left hand (first row) and the right hand (second row)

|  |  | HC_1_ | HC_2_ | HC_3_ | HC_4_ |
| --- | --- | --- | --- | --- | --- |
| PEAK AMPLITUDE (N) | Left | 7.28 ± 0.29 | 7.20 ± 0.28 | 7.56 ± 0.30 | 6.64 ± 0.27 |
|  | Right | 5.92 ± 0.26 | 6.36 ± 0.30 | 6.00 ± 0.30 | 6.16 ± 0.28 |
| PEAK TIME (s) | Left | 2.21 ± 0.09 | 2.07 ± 0.09 | 2.05 ± 0.08 | 1.94 ± 0.07 |
|  | Right | 2.26 ± 0.10 | 2.10 ± 0.09 | 2.16 ± 0.09 | 2.22 ± 0.09 |
| CV ADJUSTMENT | Left | 0.10 ± 0.01 | 0.10 ± 0.01 | 0.10 ± 0.01 | 0.14 ± 0.01 |
|  | Right | 0.13 ± 0.01 | 0.13 ± 0.01 | 0.13 ± 0.01 | 0.14 ± 0.01 |
| STD FORCE ADJUSTMENT (N) | Left | 0.69 ± 0.05 | 0.69 ± 0.05 | 0.75 ± 0.06 | 0.94 ± 0.05 |
|  | Right | 0.73 ± 0.05 | 0.76 ± 0.06 | 0.75 ± 0.06 | 0.88 ± 0.07 |
| ADJUSTMENT DURATION (s) | Left | 4.46 ± 0.45 | 4.90 ± 0.70 | 4.53 ± 0.53 | 5.21 ± 0.55 |
|  | Right | 4.42 ± 0.46 | 4.86 ± 0.69 | 4.43 ± 0.49 | 4.94 ± 0.56 |

**Table 4.** F and p values of the significant ‘Force strategy parameters’ in *Experiment 2*

|  | HAND | | TARGET FORCE | | HAND CONFIGURATION | |
| --- | --- | --- | --- | --- | --- | --- |
|  | F(1,24) | P | F(1,24) | p | F(3,72) | P |
| PEAK AMPLITUDE | 9.52 | 0.005 | 483.35 | <0.001 | 2.50 | 0.066 |
| PEAK TIME | 5.43 | 0.028 | 183.13 | <0.001 | 3.55 | 0.019 |
| CV ADJUSTMENT | 14.43 | 0.001 | 6.13 | 0.021 | 7.35 | 0.001 |
| STD FORCE ADJUSTMENT | 0.37 | 0.548 | 49.42 | <0.001 | 10.10 | <0.001 |
| ADJUSTMENT DURATION | 5.20 | 0.032 | 14.20 | 0.001 | 2.58 | 0.060 |

**Concluding remarks**

The results of this additional analysis suggest that in *Experiment 1* the loading conditions influence the kinematic strategy during the position matching task and this may cause the changes in the bimanual position performance observed in the experiments. Moreover, in *Experiment 2* the force strategy appeared to be ~~not~~ unaffected by symmetric/asymmetric proprioceptive hand configurations, but by handedness or hand preference. This strategy might be an additional reason for the independence of the accuracy of the bimanual force performance from the symmetric/asymmetric arm configurations.

Specifically, for the bimanual position sense, the movements’ strategies support the following considerations:

- in symmetric loading conditions, with both hands holding equal weights, the speed of the movements was influenced by the heaviness of these weights;
- in asymmetric conditions when the two hands, in the same trial, hold different weights, the difference between hands’ speed due to the weights decreases. Most features of the movement converge toward intermediate values with respect to those observed when both hand hold the same weights (see Table 1). This is in accordance with the inter-limb interference reported in the *Discussion*;
- the hand holding lighter weight in the asymmetric conditions has a delayed peak in the speed profile. This supports the main result that, in this case, the mismatch in the proprioceptive inputs results in a difficulty for the subjects in performing the task, as also highlighted by the residual *bias-error* in the steady-state phase.

As for the accuracy of the bimanual force control, there was a difference in all parameters depending on the hand: the left hand had a higher force peak and longer adjustment phase. These features support the hypothesis of a leading role in force control for this hand. The difference between the configurations confirm that there was not a main effect of the asymmetric/symmetric hand configuration. The other differences found with respect to the arm configurations, were more difficult to interpret when trying to relate them to the main results or deriving further conclusions. As expected, also the force exertion strategies highlighted difference due to the required target forces.

**FIGURE LEGEND**

**Figure A1. Example of speed and force profile.** The two panels show an example of (a) speed profile from the *Experiment 1* and (b) force profile from the *Experiment 2* for a single subject. Panel (a) the yellow area highlights the ballistic phase, which initiated when the speed exceeds the threshold of 10% of the first peak (horizontal dashed line) and terminates when it crosses downward which started when the speed exceeded the relative threshold. The gray area indicates the adjustment phase, which started at the end of the ballistic movement and ended with the holding time. In panel (b) the green area highlights the first part of the force profile, characterized by the main force peak. This is followed by the adjustment phase (gray area) where the mean value is indicated by a horizontal dashed line and the standard error by a dashed and dotted line.

1. Average value computed over all the single relative thresholds [↑](#footnote-ref-1)
