# Supplementary figures and images for: Interaction between position sense and force control in bimanual tasks
Source: J Neuroeng Rehabil. 2019 Nov 8;16:137. doi: 10.1186/s12984-019-0606-9 (PMC6839077; doi:10.1186/s12984-019-0606-9)

**a)**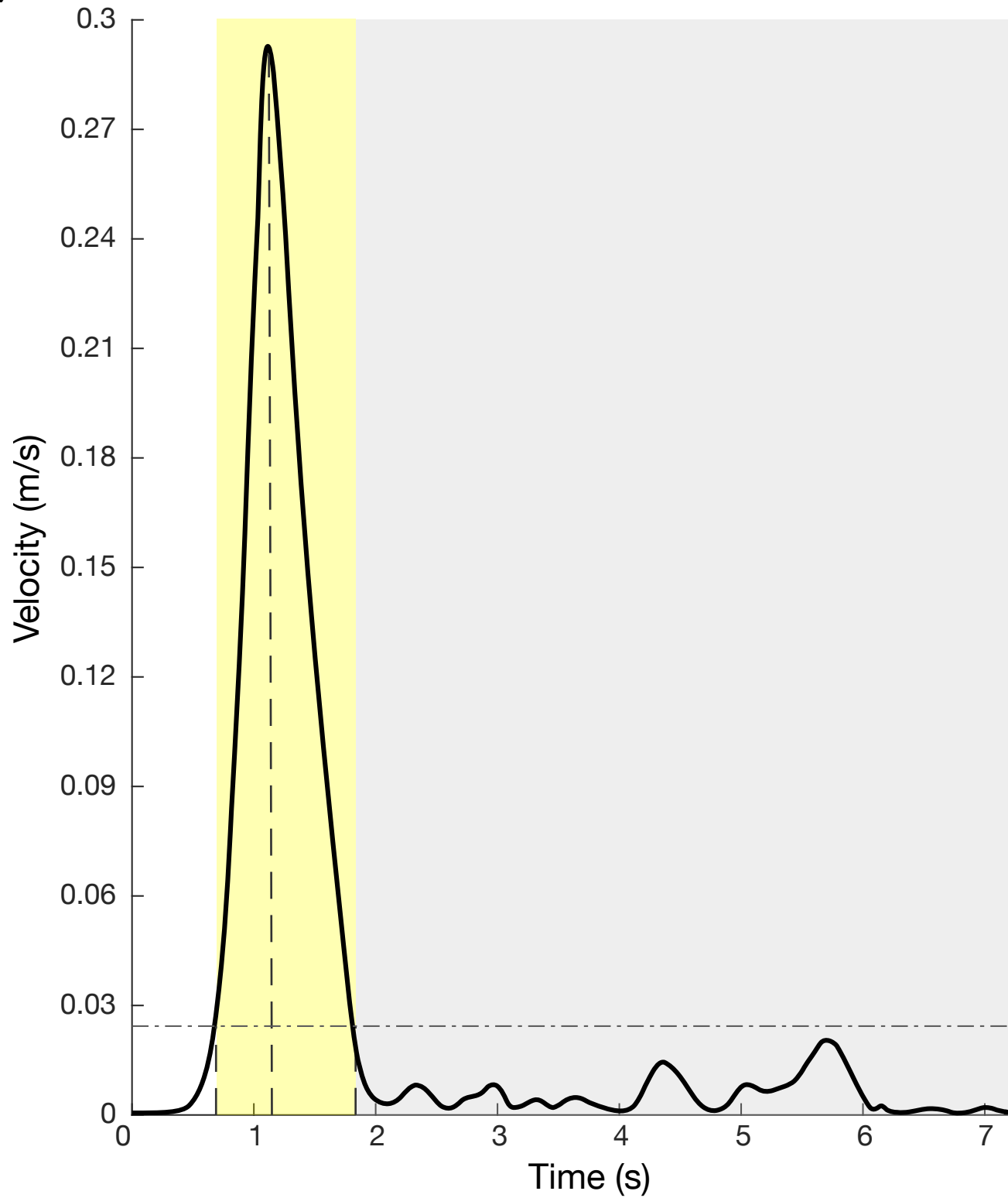**b)**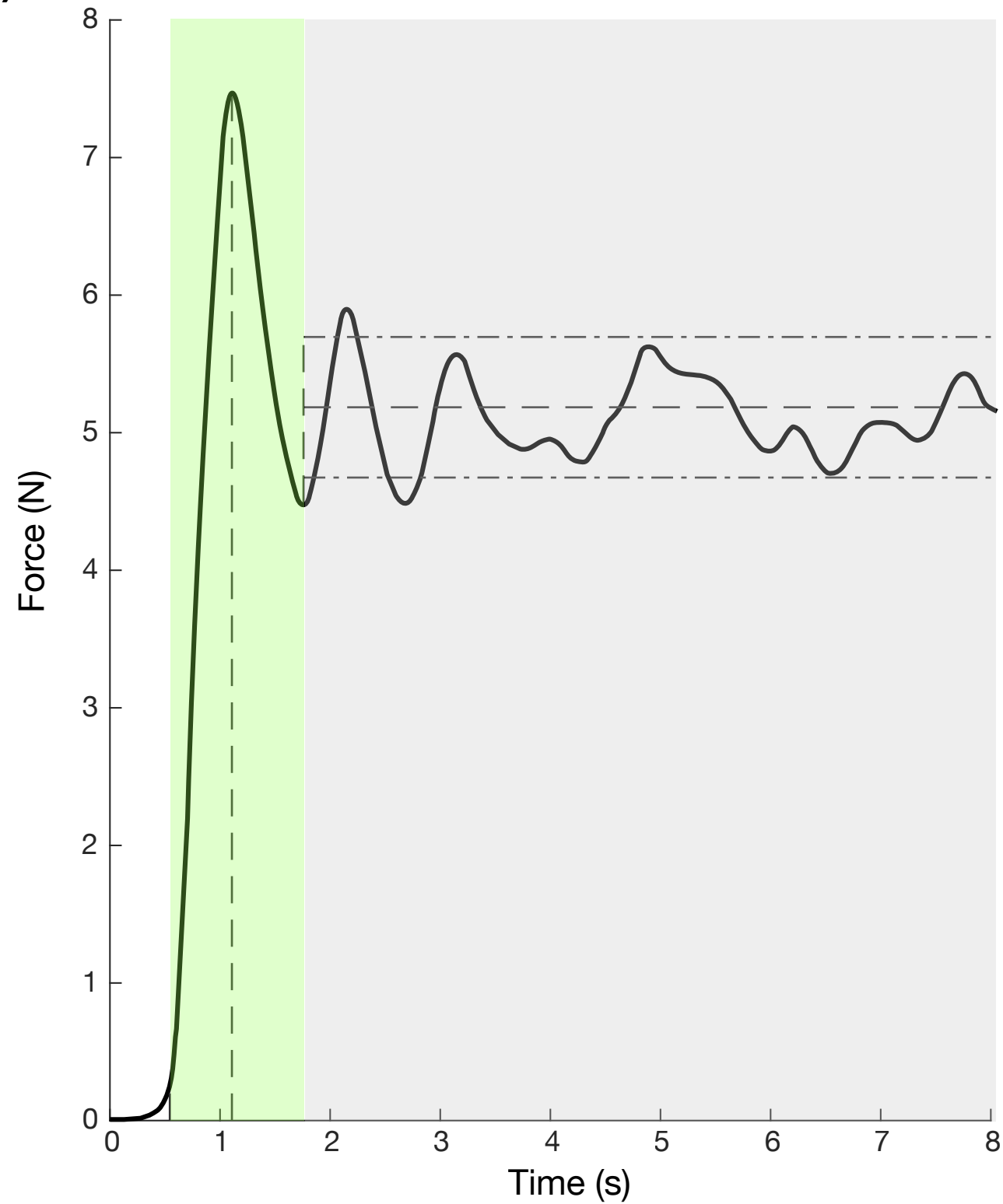

Supplement: Supplementary file 1 — Additional file 1. Analysis of the movement and force strategies applied to solve the task. We analyzed the strategies used by the subjects for accomplishing the tasks, to verify if they can provide further explanations of the results presented in the manuscript. In Experiment 1 we found that the loading conditions influenced the kinematic strategy during the position matching task. In Experiment 2 the strategy adopted for bimanual force exertion was not influenced by symmetric/asymmetric arm configurations, but by handedness or hand preference effects. Figure S1. Example of speed and force profile. [file 12984_2019_606_MOESM1_ESM.zip › 12984_2019_606_MOESM2_ESM.pdf]
